# A Retrospective Observational Study of Presentations to an Australian Emergency Department for Injuries Sustained in Exercise Pursuits Over 14 Years

**DOI:** 10.31486/toj.19.0059

**Published:** 2020

**Authors:** Robert M. Eley, Danielle Hatt, Simon Baarbe

**Affiliations:** ^1^ Emergency Department, Princess Alexandra Hospital, Brisbane, Queensland, Australia; ^2^ The University of Queensland Faculty of Medicine, Brisbane, Queensland, Australia; ^3^ The University of Queensland Faculty of Medicine, Ochsner Clinical School, New Orleans, LA

**Keywords:** *Athletic injuries*, *emergency service–hospital*, *exercise*, *physical fitness*

## Abstract

**Background:** Concurrent with the increase in the number of local gyms and the number of people engaged in fitness pursuits, exercise-related emergency department (ED) presentations have also increased. Identifying these injuries and the associated activities and equipment will help inform prevention strategies and potentially reduce the burden on the healthcare system.

**Methods:** We reviewed the presentations to an Australian tertiary hospital ED resulting from running/jogging and gym-based exercise from 2005 to 2018.

**Results:** From more than 750,000 ED visits, we identified 1,402 exercise-related presentations. Approximately two-thirds of the patients were males. Nontrauma such as chest pain and shortness of breath accounted for 11% of the presentations. Running and jogging contributed 47% of the total presentations, followed by combat activities (boxing and martial arts) with 31% of the total presentations. In the latter group, most injuries were to the head (25%) and upper limbs (39%). Injuries associated with weights/resistance activities (n=94) and falls from treadmills (n=49) accounted for 55% of the 260 injuries from use of noncombat-sports-related gym equipment. Twenty-three percent of all presentations arrived by ambulance, and overall, 9% of presentations required hospital admission. Over a 14-year period, the annual presentations rate rose from <1 to >2.5 per 1,000.

**Conclusion:** Although the annual rate of presentations to the ED from exercise has more than doubled, exercise-related presentations still constitute only a small proportion of total presentations. Nevertheless, any reduction would be advantageous to an already overstretched health system. Risk awareness and effective education about equipment and its use at point of sale and in gyms could potentially prevent many presentations.

## INTRODUCTION

Physical inactivity has been identified as the fourth leading risk factor for global mortality.^1^ In 2018, the Australian government reported that almost two-thirds (63%) of adult Australians and more than one-quarter (28%) of children >5 years of age were overweight or obese.^2^ The combined risk factors of insufficient physical activity with overweight or obesity was estimated to account for 9% of the total disease burden in Australia, a burden which is exactly equal to that attributed to the leading single risk factor of tobacco smoking.^2^ In 2011, 33.6% of the 9% disease burden from inactivity was attributed to coronary heart disease, and dementia, diabetes, bowel cancer, and stroke accounted for between 10% and 20% of the total disease burden.^3^

Government programs were initiated in response to these alarming statistics. For example, in 2018, the Australian Sports Commission started the Sport Australia Move It initiative and launched the National Sport Plan to 2030.^4^ The numerous gyms and fitness centers that have opened in recent years also encourage physical activity. Between 2005 and 2013, a 32% increase of Australians using public fitness services has been reported,^5^ and annual growth in the number of facilities between 2014 and 2019 was reported to be 5.7%.^6^ External drivers to increase exercise, such as health consciousness, are facilitated by the local availability and affordability of gyms and by promotion through social media.^6^

With exercise comes a small risk of exercise-related injury or illness. Although the epidemiology of sport injuries, and particularly team sport injuries, has been documented,^7-9^ including in Australia,^10^ literature evaluating the epidemiology of fitness activity–related injury is scarce. The few studies that exist are specific to location or equipment. These studies include gym participants who reported their injuries^11^ and emergency department (ED) presentations for injuries from weight training,^12^ treadmills,^13^ and combined martial arts and combat sports.^14^

To our knowledge, Australian studies that describe non–team sport injuries presenting to the ED are limited to one body of work.^15,16^ The studies combined common gym activities and identified the causes and types of injuries sustained at fitness facilities in the state of Victoria. Overexertion, crush injuries, falls, and awkward landings were major contributors to injuries, with weight training and group exercise being the principal activities.

Our study, undertaken in Queensland, reviewed presentations to the ED over a 14-year period. Our objective was to identify the activities and equipment that could be targeted for risk reduction and injury prevention.

## METHODS

This study was conducted in the ED of the 700-bed, tertiary-level, adult-only Princess Alexandra Hospital in Brisbane, Queensland, Australia. The annual number of presentations to the ED increased from 42,716 to 64,820 during the period under review (2005 to 2018).

The patient presentation records contained in the Emergency Department Information System dataset for 2005 to 2015 and in the FirstNet (Cerner Millennium) dataset for 2016 to 2018 provided the data for the study. Within the datasets, each patient ED presentation is registered by the triage staff. Data are collected on age, sex, arrival date and time, triage category, mode of arrival, presenting complaint, presenting problem description, primary discharge diagnosis, and discharge destination. Some of the fields are automated (eg, date and time of arrival), others are completed by selections from dropdown menus (eg, sex), and presenting complaint is entered as free text by the triage nurse based on the nurse's observations and information provided by the patient. The triage nurses in the hospital are experienced in asking and recording information about the activity and mechanism of injury, as this information is helpful for diagnosis and clinical management. Furthermore, the nurses operate within a research culture and are trained to provide as much detail as possible in the limited time they have available for documentation.

The combined 14-year dataset contained in excess of 750,000 patient records that required initial screening for inclusion into the analysis arm of the study. We used the free-text presenting complaint field for the initial screening via a targeted keyword search. All patient entries that included any of the keywords listed in Table 1 were extracted. Variations of these keywords and wildcards were included to account for spelling errors. The keywords were identified from the literature, from consultation among the researchers, from colleagues, and from review of ED triage notes.

**Table 1.** Search Words and Terms Used to Identify Cases

Aerobics	Jiu jitsu	Run
Barbell	Jog	Spar
Bench press	Judo	Squats
Boot camp	Karate	Tae kwon do
Box	Krav Maga	Jujitsu
Cross fit	Kung fu	Thai boxing
Cross training	Lunges	Taekwon-do (TKD)
Dead lifts	Martial arts	Train
Dumbbell	Muay Thai	Treadmill
Elliptical trainer	Mixed marital arts (MMA)	Weights
Exercise	Pilates	Weight lifting
Exercise bike	Pole dancing	Workout
Fitness	Resistance	Yoga
Gym	Row	Zumba

The targeted keyword search identified 7,260 presentations. We manually reviewed these records by reading the presenting complaint free text. All records that were not related to voluntary exercise (eg, “running across road to catch bus,” “driving to fitness class”) were excluded. Further review removed entries resulting from participation in a team sport activity such as rugby, cricket, or soccer. Presentations resulting from exercise undertaken as part of stress tests or cardiac rehabilitation were also excluded, as were those for which a prior cardiac event was reported. All 3 authors reviewed the resulting list for consensus on which cases should be eliminated because the exercise was incidental to the presentation. For example, an entry of “Jaw pain onset post waking radiating to left shoulder. Well last night. Persisted through yoga class” was discarded, as was “Injury right foot, tripped while running in garden in flip-flops at 1030 hrs. Now foot swollen, unable to weight-bear. Nil bruising.” Following the manual review of the records, 1,402 patient records remained for analysis.

Records were sorted into 7 categories: yoga, group exercise (including Zumba and water aerobics), resistance and weight training, treadmill, other gym equipment (eg, stepper, stationary bike, unspecified), combat and martial arts (eg, boxing, jujitsu, karate), and running (including jogging but excluding treadmill). We identified the injured body part or medical condition from the presenting problem text and the primary diagnosis from the treating doctor's entry. Descriptive statistics were generated using SPSS Statistics v.25 (IBM Corp.).

Ethics approval for this study was granted by the Human Research Ethics Committee (EC00167), application number HREC/15/QPAH/832. Data release was approved by the Health Innovation, Investment and Research Office of the Government of Queensland under the conditions of the Public Health Act 2005.

## RESULTS

Two-thirds (67.05%) of the presentations were by males whose ages ranged from 16 to 83 years (Table 2). The oldest person, an 86-year-old female, presented with symptoms of vertigo and nausea after exercising at the gym and was diagnosed with dehydration. The next oldest was an 83-year-old male who fell off a treadmill and fractured his nose.

**Table 2.** Demographics and Injury Location by Activity Type, 2005-2018

		Demographics		Injury Location/Type				
Exercise Category	n, %	Male, n (%)	Age Range, years	Head, n (%)	Upper Limb, n (%)	Lower Limb, n (%)	Torso, n (%)	Nontrauma, n (%)
Yoga	15 (1.07)	3 (20.00)	16-70	4 (26.67)	3 (20.00)	3 (20.00)	1 (6.67)	4 (26.67)
Group exercise^a^	23 (1.64)	4 (17.39)	16-66	0 (0.00)	4 (17.39)	11 (47.83)	2 (8.70)	6 (26.09)
Resistance and weight training	94 (6.70)	73 (77.66)	18-60	7 (7.45)	41 (43.62)	9 (9.57)	35 (37.23)	2 (2.13)
Treadmill	49 (3.50)	26 (53.06)	16-83	4 (8.16)	11 (22.45)	17 (34.69)	5 (10.20)	12 (24.49)
Other gym equipment^b^	117 (8.35)	78 (66.67)	18-86	12 (10.26)	14 (11.97)	32 (27.35)	35 (29.91)	24 (20.51)
Combat and martial arts^c^	441 (31.46)	366 (82.99)	16-62	111 (25.17)	172 (39.00)	99 (22.45)	53 (12.02)	6 (1.36)
Running and jogging	663 (47.29)	390 (58.82)	16-83	62 (9.35)	78 (11.76)	373 (56.26)	44 (6.64)	106 (15.99)
Total	1,402 (100.00)	940 (67.05)		200 (14.27)	323 (23.04)	544 (38.80)	175 (12.48)	160 (11.41)

^a^Includes Zumba and water aerobics.

^b^Includes stepper, stationary bike, and unspecified equipment.

^c^Includes boxing, jujitsu, and karate.

Note: Percentages are calculated across rows.

Almost half of the presentations (n=663) resulted from running or jogging. Of these, 106 (15.99%) were classified as nontraumatic, with patients complaining of chest pain, shortness of breath, nausea, dizziness, or headache. More than two-thirds of trauma cases from running and jogging involved injury to the limbs, predominately the lower limbs, as the result of ankle rolls, painful knees, and muscle strains. Only 6 cases required admission into the hospital, all of which were for wrist fractures.

Combat and martial arts training resulted in almost one-third of the presentations. Of these, 64.17% presented with injuries to the head or upper limb. Only 6 of the 441 presentations in this category were nontraumatic.

Of the 298 noncombat gym-based activities, weight and resistance training (n=94) contributed approximately one-third of the presentations, with muscle strains, sprains, and crush injuries predominating. Treadmill was the single item of identified gym equipment causing the most injuries (n=49). Almost one-quarter of the treadmill-related presentations were for nontraumatic causes, principally chest pain. During the 14-year study period, only 15 presentations were attributed to yoga and 23 to group keep-fit exercise activities.

Table 3 and the Figure depict exercise-related presentations grouped into 3 categories: gym, combat, and running. Overall, the combined exercise presentations accounted for fewer than 2 per 1,000 ED presentations. However, over the 14-year period, this rate increased from <1 per 1,000 to >2.5 per 1,000 presentations. Across the 3 categories, the rate of running presentations doubled, and gym and combat presentations almost quadrupled.

**Table 3.** Exercise Injuries from Gym, Combat, and Running Activities and Rates per 1,000 Emergency Department (ED) Presentations, 2005-2018

Year	ED Presentations, n	Gym, n	Gym/1,000 Presentations	Combat, n	Combat/1,000 Presentations	Running, n	Running/1,000 Presentations	Total	Total/1,000 Presentations
2005	42,716	5	0.12	11	0.26	25	0.59	41	0.96
2006	43,222	9	0.21	23	0.53	27	0.62	59	1.37
2007	45,727	9	0.20	20	0.44	27	0.59	56	1.22
2008	46,527	15	0.32	17	0.37	27	0.58	59	1.27
2009	46,014	13	0.28	25	0.54	27	0.59	65	1.41
2010	47,305	11	0.23	35	0.74	37	0.78	83	1.75
2011	53,698	11	0.20	32	0.60	38	0.71	81	1.51
2012	57,297	14	0.24	28	0.49	36	0.63	78	1.36
2013	59,820	19	0.32	39	0.65	52	0.87	110	1.84
2014	61,921	32	0.52	39	0.63	63	1.02	134	2.16
2015	61,991	33	0.53	33	0.53	69	1.11	135	2.18
2016	60,154	44	0.73	38	0.63	85	1.41	167	2.78
2017	62,865	50	0.80	39	0.62	75	1.19	164	2.61
2018	64,820	33	0.51	62	0.96	75	1.16	170	2.62
Total	754,077	298	0.40	441	0.58	663	0.88	1,402	1.86

**Figure.**
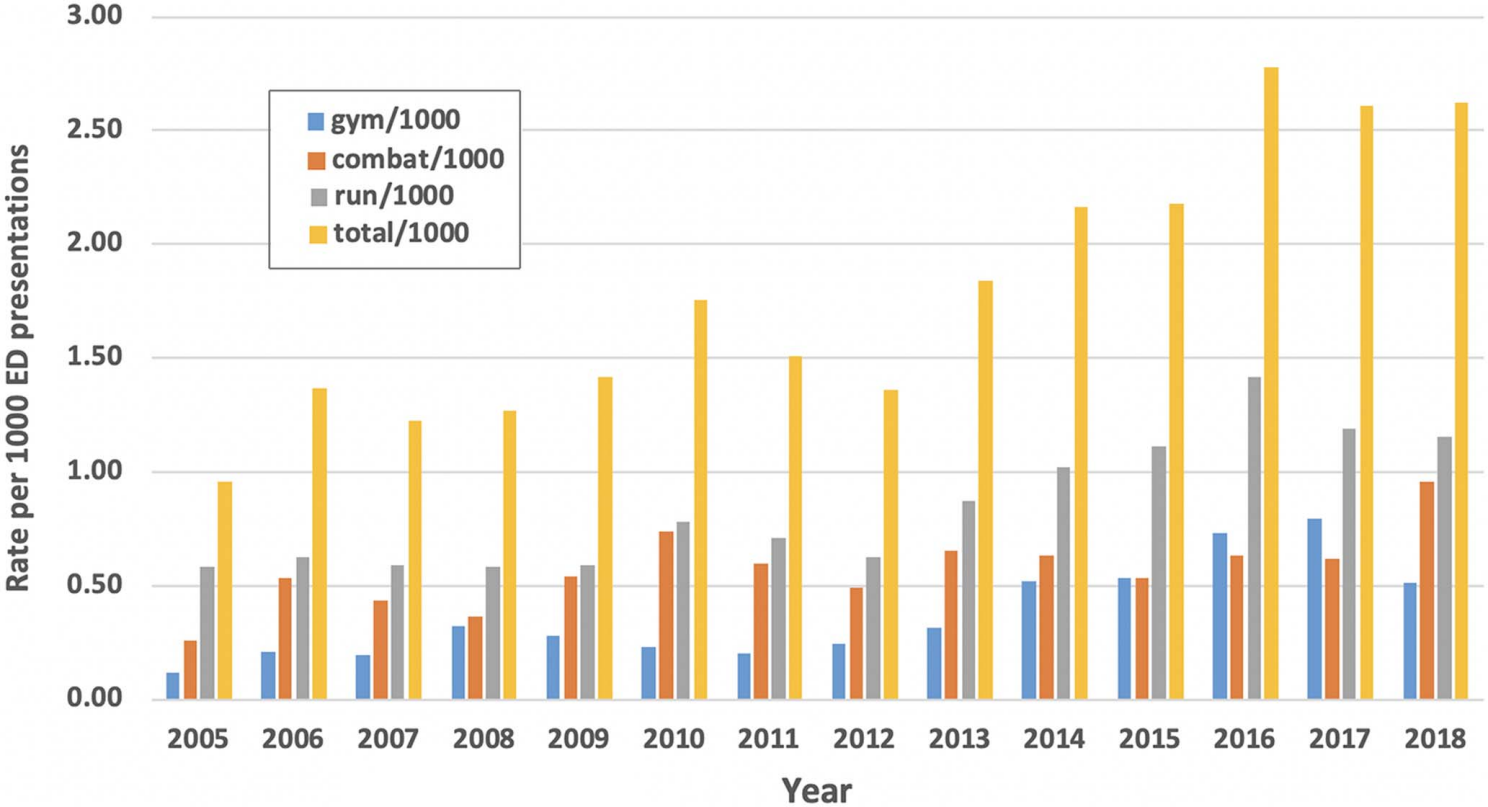
**Exercise presentations per 1,000 emergency department presentations.**

Almost one-quarter of presentations (325, 23.18%) arrived by ambulance, and of the 1,402 presentations, 105 (7.49%) went to short stay for periods of up to 12 hours before discharge, with a further 127 (9.06%) requiring in-patient hospital admission.

## DISCUSSION

The only previously reported Australian studies with a similar set of data are those by Gray and Finch from Victoria, Australia.^15,16^ We followed their lead and excluded team sport from our study. Our work adds to the literature with a patient population from Queensland, Australia.

The Victorian data identified free weights, group exercises, treadmill, and boxing as the leading activities causing injuries. Our study is in general agreement with respect to free weights, treadmills, and combat, although Gray and Finch make no reference to combat sports other than boxing. On the other hand, our data revealed few presentations related to group-class exercises; however, this difference could be because the Victorian study grouped all gym equipment under group exercise.

The body location of the injuries was largely as would be expected, with running injuries predominately to lower limbs from sprains and strains to knees and ankles and to the head from falling. Similarly, free weights were associated with upper limb and torso injuries caused by falling weight or being trapped by weights. Similar to the Gray and Finch studies, the nontraumatic presentations in our study were related to overexertion or strenuous activity.

Overall, the rate of presentation as a reported result of exercise was low, with fewer than 2 per 1,000 presentations. This rate equates to 2 presentations every week to the study ED. Compared to team sport presentations to our ED, this exercise activity rate is lower, as an unpublished audit of injuries from soccer, rugby, and cricket for 2017 showed a rate of 5.1 per 1,000 presentations.

The most recently published Australian sport and recreation participation data identify fitness and gym-related activities, after walking, to be the most popular physical activities.^17^ Owing to changes in classification, participation time series data for gym-based activities are not available. However, government industry statistics note that clubs have undergone “exceptional growth over the past five years attracting new customers with affordability and accessibility.”^18^ Since 2005, the number of fitness centers and gyms has increased by 50%, with the most significant area of growth in the availability of 24-hour clubs.^19^ Furthermore, market research data project large increases in home-based fitness equipment sales for the United States and Europe.^20^ No evidence suggests that similar increases will not be seen in Australia. These facts not only support the quadrupling of ED presentations that we showed in this study, but they also support the prediction of further increases in the future.

Jogging and running are third in participation rate, and time series data are available for these activities, with participation rates doubling from 2005-06 to 2011-12.^17^ In our data, running-related presentations to the ED also doubled and thus demonstrate good alignment with participation rates.

Combined martial arts and boxing are reported to be the 11th most popular activities in Australia, and from 2005-06 to 2013-14, participation increased 20%.^17^ This 20% increase for those activities is far less than that suggested by our ED presentations that rose 350% over the same time period. This discordance may reflect a combination of the changes in recent years in the locations where these activities occur, their governance, and the types of participants engaging in them. Combat activities have changed from being predominately standalone and organized sports with participants registered with their own governing body to being incorporated into nonsport activities that are components of high-intensity workout sessions in regular gyms. Activities such as Bootcamp and F45 Training are promoted across popular media with headlines such as “Boxing Workouts for Weight Loss.”^21^

The rates of presentations to our ED, when compared to statewide rates of participation reported by the government,^17^ indicate that injuries are more prevalent in combat sports, followed by running and then fitness/gym. The number of nontraumatic injuries in the gym environment was fewer than 4 per year, and the combat sport category had only 6 in total. We speculate that the requirement for premembership disclosure, induction processes, and the use of personal trainers could be factors.

Government statistics show increases in exercise participation rates. Whether the government initiatives to improve health through exercise are successful at a national level remains to be seen. Locally, our results indicate that while ED presentations from exercise constitute only a small proportion of total presentations, they are increasing. Consequently, any reduction in those presentations would be advantageous to an already overstretched health system. Our results also indicate that many presentations are associated with exercise equipment. Effective education about safe equipment use may prevent some presentations. More awareness of the small but potential risks of injury associated with home gyms, weights, and treadmills should be promoted at purchase, and education at gym orientation sessions could be improved. Anecdotally, as experienced by the senior author, sales and orientation are seldom accompanied by advice or guidance.

Our study is limited by its reliance on the free text provided by the triage nurses, and the specific activity undertaken when the injury occurred was sometimes missing. Because of the sheer volume of cases, retrieving more information on many patients was not only impractical but also impossible, as files had been archived or destroyed. Except for running/jogging, the location where the activity was taking place was sometimes not reported. Consequently, we were unable to report how many injuries occurred in gyms and clubs vs at home. Some items of gym equipment were also not reported; however, there is no reason to suggest that nonreporting was biased towards any type of equipment. Similarly, we were unable to report whether the activity was sport or nonsport. The former is defined as physical activity that, by its nature, is organized and has a sport governing body.^22^ However, as combat activities are increasingly undertaken in fitness gyms or at home where they are not under the auspices of a governing body, this definition has somewhat lost its meaning. Finally, although patients with chest pain and a known prior related medical condition were excluded from analysis, some of these cases were possibly not reported and thus missed and included in the analysis.

## CONCLUSION

This study demonstrated that ED visits resulting from fitness activities rose during a 14-year period (2005 to 2018). While running/jogging injuries contribute the largest burden, combat-type activities contribute the highest rate by participation. Despite the presentation rate of exercise injuries in 2018 still being low at 2.6 per 1,000 presentations, any reduction would benefit the already-stretched ED resources. Risk awareness and effective education about safe equipment and its use at point of sale and in gyms could potentially prevent some presentations.

## Notes

This article meets the Accreditation Council for Graduate Medical Education and the American Board of Medical Specialties Maintenance of Certification competencies for Patient Care, Medical Knowledge, and Practice-Based Learning and Improvement.
